# Medical College Notes

**Published:** 1895-03

**Authors:** 


### Medical College Note.

MESSRS. FREDERICK STEARNS & Co., of Detroit, have established a $600 fellowship in the University of Michigan, to be known as the Stearns Fellowship of Pharmaceutical Chemistry and Pharmacology. The object is to afford opportunity to graduates of pharmacy for original work. During the coming year the work of the candidate will be under the immediate supervision of Dr. A. B. Prescott, dean of the department of pharmacy.

